# Hospital Malnutrition in the Medicine and Neurology Departments: A Complex Challenge

**DOI:** 10.3390/nu15245061

**Published:** 2023-12-11

**Authors:** Erica Starace, Giulia De Pasquale, Emanuela Morenghi, Camilla Crippa, Sofia Matteucci, Gabriella Pieri, Fanny Soekeland, Stefano Maria Gibbi, Giuliana Lo Cricchio, Francesco Reggiani, Marta Calatroni, Manuela Pastore, Beatrice Mazzoleni, Stefano Mancin

**Affiliations:** 1 IRCCS Humanitas Research Hospital, 20089 Milan, Italy; erica.starace@humanitas.it (E.S.); giulia.depasquale@humanitas.it (G.D.P.); emanuela.morenghi@humanitas.it (E.M.); camilla.crippa@humanitas.it (C.C.); sofia.matteucci@humanitas.it (S.M.); gabriella.pieri@humanitas.it (G.P.); giuliana.locricchio@asst-santipaolocarlo.it (G.L.C.); francesco.reggiani@hunimed.eu (F.R.); marta.calatroni@hunimed.eu (M.C.); manuela.pastore@humanitas.it (M.P.); 2 Department of Biomedical Sciences, Humanitas University, 20072 Milan, Italy; beatrice.mazzoleni@hunimed.eu; 3 School of Health Professions, University of Applied Sciences, 3008 Bern, Switzerland; fanny.soeke@gmail.com; 4 Department of Drug Science, School of Pharmacy, University of Pavia, 27100 Pavia, Italy; stefanomaria.gibbi01@universitadipavia.it

**Keywords:** malnutrition, food intake, nutritional assessment, systematic review

## Abstract

Hospital malnutrition is especially common among elderly patients with neurological deficits or dementia. These conditions can be exacerbated by unpalatable diets and issues such as dysphagia and presbyphagia. Our study aimed to investigate the prevalence of malnutrition in patients on a homogenized diet and to identify potential correlations with specific clinical variables. We conducted a retrospective observational study in compliance with the STrengthening the Reporting of OBservational studies in Epidemiology (STROBE) guidelines. The study encompassed 82 patients, mainly elderly and diagnosed with neurodegenerative diseases. Upon initial assessment, 46.34% of the sample displayed a risk of malnutrition based on the Malnutrition Universal Screening Tool (MUST), and 62.20% were classified as malnourished based on the Global Leadership Initiative on Malnutrition (GLIM) criteria. Only 45.12% retained autonomy in food intake. Weight loss identified prior to the study was closely tied to malnutrition and influenced BMI. Moreover, autonomy in food intake was strongly associated with a prolonged hospital stay (LOS), and a similar trend was observed for water intake. Our findings emphasize the importance of promptly recognizing patients at risk of malnutrition, especially within such a vulnerable population. Autonomy in food intake and hydration emerge as critical indicators in the clinical management of hospitalized patients.

## 1. Introduction

Hospital malnutrition represents a complex and critical challenge in medicine and neurology. Severe protein-calorie malnutrition has been observed in 20–50% of hospitalized patients [1,2]. This population frequently experiences feeding difficulties due to various factors, including neurological deficits, dementia, dysphagia, and age-related problems such as presbyphagia and edentulism [1,2,3,4]. Moreover, neurological deficits significantly impede adequate nutrition in hospitalized patients. In the case of paralysis or impaired voluntary movements, patients may experience considerable difficulty in handling food and swallowing. This condition often occurs in patients with neurodegenerative diseases, such as Parkinson’s disease, stroke, or traumatic brain injury. Restricted movements and reduced muscle coordination make it difficult to chew and swallow food, seriously compromising nutritional intake [2].

Dementia, particularly Alzheimer’s disease, represents another significant cause of feeding difficulties in medical and neurological settings. Patients with dementia may experience cognitive difficulties, disorientation, and memory loss. These conditions can affect their ability to feed themselves and maintain regular meal patterns. Consequently, malnutrition becomes a relevant issue for these vulnerable patients [2,5].

Dysphagia, a condition characterized by swallowing difficulties, is another determining factor in hospital malnutrition [6,7,8,9]. The reported incidence of dysphagia in people 65 years of age and older is 7–13%, while the prevalence rates increase above 30% in patients with neurodegenerative diseases [6,8,9,10]. Dysphagia can be caused by muscle problems, such as esophageal or gastrointestinal dysfunction, or neurological factors, such as central nervous system lesions [6,7,8,10]. Patients with dysphagia face significant challenges in swallowing solid and liquid foods, increasing the risk of an inadequate diet and the danger of aspiration pneumonia [6,7,8,9,10,11].

Age-related issues such as presbyphagia, a condition referring to swallowing difficulties, and edentulism, the lack of teeth that can affect chewing ability and effective food breakdown, further contribute to the feeding difficulties encountered in hospitals. These age-related factors require modifications in food consistency and dietary approaches to ensure adequate nutrition for affected patients [7,9].

Furthermore, it is essential to emphasize that these underlying conditions, characterized by neurological deficits, dementia, dysphagia, and age-related issues, are often associated with various comorbidities that can influence nutrition and nutritional status [12,13]. Chronic conditions such as diabetes, hypertension, cardiovascular diseases, and chronic obstructive pulmonary diseases are the most common comorbidities, negatively impacting appetite, nutrient assimilation, and utilization, thereby increasing the risk of malnutrition [2,9]. Additionally, acute states necessitating hospitalization can significantly influence the nutrition of elderly patients. Acute infections such as pneumonia or urinary tract infections can cause an increase in metabolism and energy requirements that may not be adequately met due to reduced or compromised food intake [14]. Prolonged immobility and dietary restrictions resulting from acute events, such as stroke or fall-related fractures, can negatively impact the feeding and nutrition of elderly patients, increasing the risk of malnutrition [15]. In addition to these clinical conditions, another factor that can increase the risk of malnutrition is the hospital diet itself [16]. Often, diets in this patient population are unappetizing to patients, either due to restrictions imposed by texture modifications, such as pureed diets, or due to reduced caloric and protein content. Food blending, a common practice in hospital settings to modify the texture of meals for patients with feeding difficulties, can negatively affect palatability, making the diet less enjoyable and reducing patients’ motivation to consume adequate nutrients [17].

### Study Objective

The main objective of this study was to evaluate the prevalence and risk of malnutrition in a specific sample of patients following a homogenized diet, using the Malnutrition Universal Screening Tool (MUST) scale and the Global Leadership Initiative on Malnutrition (GLIM) criteria. A further objective of this study was to identify potential correlations between malnutrition/risk of malnutrition, variables of clinical interest, factors influencing nutritional status, autonomy in food intake, and the length of the hospital stay (LOS).

## 2. Materials and Methods

### 2.1. Study Design

This study was an observational and retrospective investigation utilizing data retrieved from the electronic medical archives of the IRCCS Humanitas Research Hospital in Rozzano, Milan. Access to these records was granted by the Medical and Health Directorate and approved by the Ethical Committee CET Lombardia 5, with authorization number 22/23. Additionally, the research methodologies adhered to the guidelines outlined in the STrengthening the Reporting of OBservational studies in Epidemiology (STROBE) [18].

### 2.2. Inclusion and Exclusion Criteria

Inclusion criteria: (1) Adult patients admitted to the Internal Medicine and Neurology departments at the IRCCS Humanitas Research Hospital of Rozzano between January and March 2023; (2) patients prescribed a homogenized diet due to feeding difficulties arising from swallowing disorders (including dysphagia), chewing difficulties, neurological problems, edentulism, or any other condition necessitating a homogenized diet; (3) no clinical contraindications to oral food intake; (4) independence in diet, unless partial independence or complete inability to self-feed, in which case patients were supported by caregivers or family members.

Assessment Criteria: Considering that several factors can influence food intake, including the level of physical activity, mental state, and cognitive function, the nursing staff assessed the levels of physical activity and the functional autonomy of the patients at the time of admission using the electronic nursing chart, which incorporates a specific section for planning and assessing care needs. Those identified with impaired self-administration of meals were cared for by the support team, which comprised auxiliary staff members responsible for meal administration. For mental status and cognitive function, we used the Glasgow Coma Scale (GCS), a clinical tool that assesses a patient’s level of consciousness, providing a score between 3 (deeply unconscious) and 15 (fully conscious); this allowed us to exclude patients with severe cognitive impairment. The presence of dysphagia, identified during hospitalization, was diagnosed through the 3OZ water swallow test. This test evaluates a patient’s ability to swallow a certain volume of water without showing signs of respiratory difficulty or choking. The degrees of dysphagia, ranging from 1 to 4, respectively, indicate mild to severe dysphagia. For our study, we included only patients with levels 2 and 3 dysphagia, corresponding to those requiring a homogeneous consistency diet specifically provided by the hospital catering service (Supplementary File S1). In this regard, the supplied diet could include bottled water or thickened water in 125 mL containers, depending on whether the patient exhibited a liquid dysphagia type or not. Patients with level 1 dysphagia, who did not present significant issues, and those with level 4 dysphagia, as they had severely compromised swallowing and might require more complex dietary interventions or alternatives to oral nutrition, were excluded from the study. In the selected patients, the diagnosis and degree of dysphagia were further confirmed by an in-depth evaluation by a speech therapist and the treating physician. 

Exclusion criteria: (1) Inability to take oral nutrition; (2) complications, such as significantly altered neurological status (Glasgow Coma Scale ≤ 8); (3) nonhomogenized diet. 

In this retrospective study, a cohort of 86 eligible participants was included based on the predefined inclusion and exclusion criteria. Given the exploratory nature inherent in this retrospective observational study, all eligible participants with a prescribed diet during the initial quarter of 2023 were incorporated into the analysis.

### 2.3. Data Collection

All the necessary information for conducting this study has been collected and analyzed retrospectively from a secure and anonymized electronic database. The data are based on standardized care provided at the hospital where the study was conducted. We conducted a retrospective analysis of data from digital medical records, strictly adhering to the predefined inclusion/exclusion criteria outlined in the study protocol. The information obtained retrospectively stems from standard procedures implemented in the enrolled hospital departments of medicine and neurology. These procedures were designed to meticulously document dietary intake through specific modules integrated into electronic medical records. These modules include the quantitative recording of meals for breakfast, lunch, and dinner, as well as snacks and daily water consumption. Furthermore, the data collected retrospectively derive from a data collection process conducted starting from the patient’s hospitalization, and are divided into the following phases:

(1) Upon hospital admission: Recording parameters such as age, gender, body mass index (BMI), plasma albumin concentration, primary diagnosis, and comorbidities (such as diabetes type I–II, cardiac conditions, chronic kidney disease stage I–IV, chronic obstructive pulmonary disease, and cancer).

(2) Within the first 72 h of admission or upon the prescription of a specific diet: Assessment of fluid and food intake according to the established protocol in our hospital, supervised by the nursing team and endorsed by medical professionals from the medicine and neurology departments. All information was documented in electronic medical records, including daily fluid intake in milliliters, consumed kilocalories, and the proportion of food ingested.

(3) Upon hospital discharge: Meticulous calculation of the entire duration of the hospital stay by analyzing admission and discharge dates and times.

This approach allowed for a comprehensive and accurate retrospective data collection reflecting the standard hospital practice applied during the study period. The collected data were subsequently utilized to meet the previously established research objectives. Given the observational and retrospective nature of the study, patients were not actively engaged, and no subsequent follow-up periods were anticipated. 

### 2.4. Study Procedures

This study comprised three main phases, commencing with a prevalence investigation to identify the following aspects within the retrospective sample: (1) assessment of malnutrition risk using the MUST scale, a five-step approach designed to identify adults at risk of malnutrition. Developed by the British Association for Parenteral and Enteral Nutrition, it involves measuring BMI, evaluating unintentional weight loss, and considering the effects of acute disease. These parameters allow the classification of an individual’s malnutrition risk as low, medium, or high. Subsequently, a care plan is established, and regular reviews are conducted to monitor patient progress [19]. (2) The presence of malnutrition was assessed using the GLIM criteria, a globally accepted standard for diagnosing malnutrition. This process includes initial screening for malnutrition risk using any validated tool, and then confirming a malnutrition diagnosis if at least one phenotypic and one etiologic criterion are met. The phenotypic criteria include unintentional weight loss, low BMI, and reduced muscle mass, while the etiologic criteria incorporate reduced food intake or absorption, and disease burden or inflammation. These criteria can be effectively applied in various healthcare settings [20]. After the initial phase focused on identifying the risk/presence of malnutrition, the study transitioned to a second phase where potential correlations between malnutrition and the risk of malnutrition were evaluated against: (1) demographic variables (age, sex); (2) clinical variables (underlying disease, comorbidity, levels of plasma albumin); (3) clinical variables that could impact nutritional status (presbyphagia, dysphagia, cognitive decline, edentulism); (4) autonomy in food intake (assessed by the nursing staff at hospital admission); (5) length of the hospital stay. Due to the retrospective design of this study, evaluations and potential correlations with complications that arose during hospitalization were considered but not applied due to the high risk of potential bias caused by previous complications, such as sepsis, pneumonia, and urinary tract infections (UTIs), that were present at the time of hospital admission or were the reason for hospital admission, and could therefore influence the observed data. For this reason, the length of stay (LOS) was considered a relevant indicator of the clinical progression of the sample, viewed as representative of possible complications that arose during hospitalization [21]. In the third phase of our investigation, we examined the association between the length of the hospital stay and (1) demographic variables (gender, age), (2) clinical variables (comorbidities, sepsis), and (3) variables related to nutritional status. This last evaluation was conducted to understand the possible impact of nutrition on recovery times and the length of hospitalization in the sample of patients studied. Specifically, a correlation was conducted with clinical variables that could influence the nutritional status: presbyphagia, dysphagia, cognitive decline, edentulism, and autonomy in food intake; food intake (kcal/day) and fluid intake (ml/day); nutritional status (albuminemia, BMI, weight loss in the previous 3–6 months); risk (MUST scale) or the presence of malnutrition (GLIM criteria).

### 2.5. Statistical Analysis

Data were described as a number and percentage, or mean and standard deviation, with a 95% confidence interval, if necessary, or median and range, as appropriated. Adherence to Gaussian distribution was verified with the Shapiro–Wilk test. Differences between groups were explored with the chi-square test, Student’s t-test, or Mann–Whitney test, as appropriated. A secondary analysis was conducted to evaluate the possible association between the scores related to the risk of malnutrition obtained with the MUST scale and demographic variables (age, sex), clinical variables (underlying disease, comorbidity, levels of plasma albumin), clinical variables that could impact the nutritional status (presbyphagia, dysphagia, cognitive decline, edentulism), autonomy in food intake, and the length of the hospital stay. A similar analysis explored the association with the presence of malnutrition measured with the GLIM criteria and the same risk factors. The last analysis searched for a possible association of the above-described risk factors and the length of stay with a linear regression analysis. All analyses were performed with Stata version 17. Significance levels were set to 0.05.

## 3. Results

### 3.1. Demographic and Clinical Characteristics of the Sample

Among the 86 eligible patients, 82 were entered in the final analysis. The final sample demonstrated a balanced gender distribution (38 males, 46.34%) and was predominantly composed of an elderly population, with an average age of 82.1 ± 10.5 years. Patients had a median hospital stay of 16 days, with durations ranging between 5 and 86 days. When evaluating the reasons for hospitalization, neurodegenerative diseases were the most prevalent, followed by sepsis and pneumonia. Cancer and urinary tract infections (UTIs) constituted a smaller portion of our sample. In our sample, a substantial number of patients had comorbidities, with cardiopathy being the most prevalent, followed by diabetes mellitus, COPD, and CKD. Since this study evaluated a cohort on a homogenized diet, the primary reasons for dietary prescription were dysphagia (41.46%) and presbyphagia (31.71%). About one in five patients exhibited cognitive deficits and edentulism (Table 1). 

### 3.2. Nutritional Status and Dietary Autonomy

The nutritional status of the sample was retrospectively assessed by analyzing the BMI values, the weight change in the preceding 3–6 months, and the plasma albumin levels. The patients considered had an average BMI value of 22.7 ± 4.3 kg/m^2^ with a median weight variation of 2.84 kg (range 0–22.5 kg). Plasma albumin levels indicated a protein deficiency, with an average value of 29.4 ±5.6 g/L.

**Figure 1:**
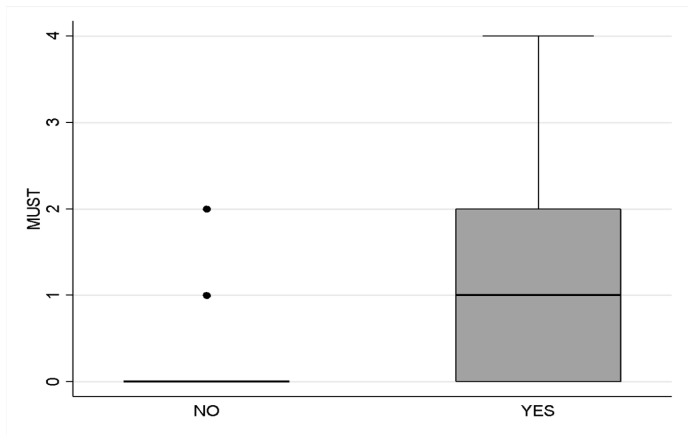
Risk and presence of malnutrition. Legend: Risk and presence of malnutrition in the sample evaluated according to the Malnutrition scale Universal Screening Tool (MUST) and the Global Leadership Initiative on Malnutrition (GLIM) criteria.

In support of these findings, the risk of malnutrition was assessed using the MUST scale upon hospital admission. A total of 53.66% of the sample scored a 0; however, a significant percentage of patients exhibited higher scores, suggesting a risk of malnutrition. Specifically, 17.07% scored a 1, 21.95% scored a 2, 2.44% scored a 3, and 4.88% scored a 4. The presence of malnourished patients was notable when evaluated based on the GLIM criteria, where 62.20% of the sample was classified as malnourished (Figure 1). Considering dietary autonomy as an important parameter, upon hospital admission, this parameter, as noted in the electronic clinical record by the nursing staff, indicated that less than half of the sample, specifically 45.12%, demonstrated autonomy in food intake. This suggests further complexities in the dietary and nutritional management of this population.

Subsequent to this initial assessment, the secondary analysis results are presented in Table 2 for the risk of malnutrition obtained with the MUST scale and in Table 3 for the presence of malnutrition measured with the GLIM criteria. The data obtained did not show a potential association between the risk or presence of malnutrition and the clinical and demographic variables considered, except for BMI, where a higher BMI had a lower risk of malnutrition, and weight loss in the previous 3–6 months, where higher weight loss had a higher risk of malnutrition (Table 2).

### 3.3. LOS, Clinical Variables, and Food and Water Intake

As a last consideration in our study, the length of the hospital stay was correlated with demographic and clinical variables, and more specifically with variables related to the homogenized diet taken and the nutritional and water intake, as well as the presence/risk of malnutrition. Analyzing the possible correlations between the LOS and “nutritional” variables, the risk of malnutrition, weight loss in the previous 3–6 months increased the LOS, while water intake and autonomy in food intake decreased the LOS. (Table 4).

## 4. Discussion

Malnutrition among hospitalized patients is a common occurrence due to various factors, including the presence of underlying diseases and comorbidities that hinder proper nutrition. However, often beyond the patient’s clinical conditions, hospital dietary regimens themselves can further exacerbate the risk of malnutrition, particularly due to modified food textures that decrease palatability and appetite [22].

The objective of this study was to assess the prevalence and risk of malnutrition in patients on a homogenized diet and to determine its correlations with their clinical status. The patient cohort predominantly included elderly individuals with neurodegenerative conditions or active infectious states, coupled with functional impairments that hampered autonomous feeding. Furthermore, these individuals generally presented with a significant array of comorbidities, predisposing them to dysphagia and presbyphagia.

Analysis of the cohort revealed that the majority were malnourished upon admission based on the GLIM criteria. Upon further nutritional risk assessment, 46.34% exhibited a tangible risk, with a score on the MUST scale greater than zero. These findings underscore a pre-existing malnourished state that could be markedly exacerbated during the hospital stay, both due to pathological conditions adversely affecting nutrition and potential autonomy loss subsequent to hospitalization, influenced by the patient’s medical condition. In fact, from the electronic medical records analyzed, only 45.12% of the cohort seemed to retain food intake autonomy. Autonomy in meal consumption proves pivotal for enhanced and increased nutrition compared to nonautonomous patients requiring comprehensive assistance. In this context, in line with recent research [22], the adoption of homogenized diets with a modified consistency not only improves the ease and practicality of taking the hospital diet, but also stimulates an increase in the daily caloric intake, attributed to the improvement in the taste and the attractiveness of the food. Consequently, a clinical assessment aimed at identifying potential dietary challenges and assessing the risk/presence of malnutrition could be complemented by the initiation of dietary or nutritional counseling. The objective is to seamlessly enhance the physician-prescribed diet by incorporating “innovative” homogenized diets tailored to specific acute and chronic conditions, including, but not limited to, diabetes, chronic renal failure, heart disease, liver disease, and others. This adaptation extends to specialized refeeding diets postsurgery, particularly after procedures involving the head–neck, the esophagogastric region, or bariatric interventions.

In subsequent evaluations, the relationship between demographic, clinical, and nutritional variables, frequently of key interest, was examined. Although subsequent analyses in the current study did not unveil direct correlations between these variables, some findings warranted deeper consideration. Specifically, the weight loss noted in the months leading up to the survey was significantly associated with malnutrition, subsequently affecting BMI. A prominent variable of interest was food intake autonomy. This parameter, meticulously evaluated by the nursing staff, revealed a strong correlation with the length of the hospital stay (LOS). Concurrently, water intake, though not precisely food intake, showcased a correlation with the LOS. These results suggest a probable link between diminished food intake autonomy, reduced water intake, and an escalated risk of malnutrition. It is conceivable to hypothesize that a lack of autonomy in food intake could lead to diminished adherence to both food and water consumption. This, in turn, might place patients at a heightened risk of malnutrition, also impacting their LOS. While our data do not explicitly show a direct link between food intake and malnutrition or the LOS, the significance of feeding autonomy and water intake stand out as pivotal factors. Given the data procured, the early identification of malnourished patients, or those at risk, is imperative to promptly implement interventions promoting improved nutrition. It would be beneficial to encourage as much feeding autonomy as is feasible, allowing patients to consume more, tailored to their pace and mode. Lastly, the adoption of more varied hospital diets, such as the modified homogenized diet (HMD), which, in a prior study [22], has been demonstrated to be more effective and favored by patients compared to standard diets, would be advised.

### Limitations

We emphasize that the present observational study has inherent limitations due to its retrospective design. Specifically, the identification of a precise and predefined sample size was not feasible, and the selection was based on the number of prescribed diets in the specific study departments. Nevertheless, despite this limitation, we believe that the study sample is representative of current hospital dietary practices, shedding light on the day-to-day scenarios faced by these departments. Additionally, it is important to highlight that the plasma albumin levels presented in our results, indicative of protein deficiency, may be influenced by various factors, such as hydration status and inflammation, along with other elements that could impact the overall accuracy of the protein status assessment.

## 5. Conclusions

This study highlights that elderly patients within the sample were often at risk of malnutrition, and the majority were indeed malnourished. Furthermore, the lack of autonomy in feeding led to a decrease in food intake, and a subsequent decline in the nutritional status and an overall clinical condition of the considered sample. Given these variables, this retrospective study suggests that further research should be directed towards the early identification of patients not only at risk of malnutrition or already malnourished, but also focusing on autonomy in primary needs, such as feeding and hydration. To this end, the adoption of diets with a modified consistency could enhance the food intake of this population segment, particularly due to the improved meal intake practicality and increased palatability.

## Figures and Tables

**Table 1:** Sample characteristics.

n	82
Sex (male) (n, %)	38 (46.34%)
Age (years) (mean, SD)	82.1 ± 10.5
BMI (kg/m^2^) (mean, SD)	22.7 ± 4.3
Length of stay (days) (median, range)	15 (5–86)
Reason for hospitalization	
Cancer	9 (10.98%)
UTIs	8 (9.76%)
Pneumonia	21 (25.61%)
Neurodegenerative pathologies	30 (36.59%)
Sepsis	22 (26.83%)
Comorbidity (n, %)	53 (64.63%)
DM (n, %)	20 (37.74%)
Cardiopathy (n, %)	27 (50.94%)
COPD (n, %)	16 (30.19%)
CKD (n, %)	16 (30.19%)
Reason for diet	
Edentulous (n, %)	15 (18.29%)
Cognitive impairment (n, %)	19 (23.17%)
Dysphagia (n, %)	34 (41.46%)
Presbyphagia (n, %)	26 (31.71%)

Legend: Data are presented as number (%), mean ± SD, or median (range). BMI: body mass index; UTIs: urinary tract infections; DM: diabetes mellitus; COPD: chronic obstructive pulmonary disease; CKD: chronic kidney disease; MUST: Malnutrition Universal Screening Tool; GLIM: Global Leadership Initiative on Malnutrition.

**Table 2:** Risk of malnutrition, clinical variables, and nutritional status.

	MUST			
	0	1	>2	*p* Value
n	44	14	24	
Sex (male)	19 (43.18%)	9 (64.29%)	10 (41.67%)	0.394
Age (years)	82.0 ± 11.4	83.4 ± 8.0	81.4 ± 10.5	0.885
BMI (kg/m^2^)	24.2 ± 4.6	23.1 ± 2.6	19.6 ± 2.6	<0.001
Length of stay (days)	13 (6–76)	23.5 (6–59)	16 (5–86)	0.332
Comorbidity	27 (61.36%)	10 (71.43%)	16 (66.67%)	0.820
Reason for diet				
Edentulous	8 (18.18%)	4 (28.57%)	3 (12.50%)	0.469
Cognitive decay	8 (18.18%)	4 (28.57%)	7 (29.17%)	0.519
Dysphagia	20 (45.45%)	6 (42.86%)	8 (33.33%)	0.642
Presbyphagia	13 (29.55%)	4 (28.57%)	9 (37.50%)	0.807
Albuminemia (g/L)	29.9 ± 5.6	30.2 ± 5.0	28.2 ± 5.9	0.426
Weight loss in the previous 3–6 months	0 (0–4.62)	5.56 (0–9.76)	10.32 (0–22.5)	<0.001
Autonomy in food intake	25 (56.82%)	4 (28.57%)	8 (33.33%)	0.073

Legend: Data are presented as number (%), mean ± SD, or median (range). SD: standard deviation; BMI: body mass index; MUST: Malnutrition Universal Screening Tool; GLIM: Global Leadership Initiative on Malnutrition.

**Table 3:** Malnutrition, clinical variables, and nutritional status.

	GLIM		
	Yes	No	*p* Value
n	51	31	
Sex (male)	23 (45.10%)	15 (48.39%)	0.772
Age (years)	83.7 ± 9.0	79.5 ± 12.4	0.079
BMI (kg/m^2^)	21.1 ± 2.6	25.3 ± 5.2	<0.001
Length of stay (days)	14 (6–86)	18 (5–76)	0.193
Comorbidity	35 (68.63%)	18 (58.06%)	0.332
Reason for diet			
Edentulous	9 (17.65%)	6 (19.35%)	0.846
Cognitive decay	14 (27.45%)	5 (16.13%)	0.289
Dysphagia	20 (39.22%)	14 (45.16%)	0.596
Presbyphagia	15 (29.41%)	11 (35.48%)	0.597
Albuminemia (g/L)	28.9 ± 5.6	30.3 ± 5.5	0.254
Weight loss in the previous 3–6 months	5.17 (0–22.5)	0 (0–15.31)	<0.001
Autonomy in food intake	22 (43.14%)	15 (48.39%)	0.643

Legend: Data are presented as number (%), mean ± SD, or median (range). SD: standard deviation; BMI: body mass index; MUST: Malnutrition Universal Screening Tool; GLIM: Global Leadership Initiative on Malnutrition.

**Table 4:** LOS, clinical variables, and food and water intake.

	Slope (95%CI)	*p* Value
Sex (male)	2.25 (−5.66; 10.16)	0.573
Age (years)	−0.45 (−0.81; −0.08)	0.016
BMI	0.73 (−0.18; 1.64)	0.115
Comorbidity	−5.79 (−13.96; 2.37)	0.162
Reason for diet		
Edentulous	−8.28 (−18.34; 1.77)	0.105
Cognitive decay	−5.35 (−14.64; 3.94)	0.256
Dysphagia	1.88 (−6.13; 9.89)	0.642
Presbyphagia	1.32 (−7.16; 9.81)	0.757
Albuminemia (g/L)	0.46 (−0.25; 1.16)	0.199
Weight loss in the previous 3–6 months	1.21 (0.47; 1.94)	0.002
Acute pathology	−11.11 (−18.82; −3.39)	0.005
GLIM	−6.03 (−14.07; 2.01)	0.139
MUST	3.43 (0.02; 6.84)	0.049
Autonomy in food intake	−22.28 (−28.49; −16.08)	<0.001
Fluid intake (100 mL)	−1.16 (−2.06; −0.25)	0.013
Food intake (100 Kcal)	−0.43 (−2.38; 1.51)	0.658

Legend: BMI: body mass index; MUST: Malnutrition Universal Screening Tool; GLIM: Global Leadership Initiative on Malnutrition.

## Data Availability

Clinical data and clinical-assistance forms are available upon request by contacting the corresponding author, and authorization from the hospital facility is required.

## Supplementary Materials

The following supporting information can be downloaded at: https://www.mdpi.com/article/10.3390/nu15245061/s1, The dietary pattern of the homogenized diet utilized in the study (weekly menu) is provided in Supplementary File S1.

## Abbreviations

BMI: body mass index; CKD: chronic kidney disease; COPD: chronic obstructive pulmonary disease; GLIM: Global Leadership Initiative on Malnutrition; IRCCS: Institute for Scientific Hospitalization and Care; IQR: interquartile range; Kcal: kilocalories; LOS: length of the hospital stay; MUST: Malnutrition Universal Screening Tool; STROBE: Strengthening the Reporting of Observational Studies in Epidemiology
