# Supplementary material for: Hospital Malnutrition in the Medicine and Neurology Departments: A Complex Challenge
Source: Nutrients. 2023 Dec 11;15(24):5061. doi: 10.3390/nu15245061 (PMC10745339; doi:10.3390/nu15245061)
Supplement: Supplementary file 1 [file nutrients-15-05061-s001.zip › nutrients-2761341-supplementary.pdf]

File S1. Dietary pattern of the homogenized diet utilized in the study (weekly menu)

LUNCH		MONDAY	TUESDAY	WEDNESDAY	THURSDAY	FRIDAY	SATURDAY	SUNDAY
First Course		Legumes cream	Rice and tomato cream	Rice and peas cream	Broccoli cream	Saffron rice cream	Semolina and green beans cream	Rice and tomato cream
Second Course		Loin mousse	Ham and ricotta mousse	White meat mousse	Ham and ricotta mousse	White meat mousse	Cod mousse with aromas	Red meat mousse
Side Dish		Broccoli mousse	Purèe	Spinach mousse	Carrot mousse	Courgette mousse	Purèe	Purèe
Dessert		Pudding	Pudding	Pudding	Pudding	Pudding	Pudding	Pudding
Energy	Kcal	838	646	891	820	859	649	617
Soluble sugar	g	16	21	14	22	15	18	19
Available carbohydrates	g	128	73	152	115	147	107	84
Lipids	g	16	17	14	22	14	10	10
Protein	g	41	24	37	35	34	30	28

DINNER		MONDAY	TUESDAY	WEDNESDAY	THURSDAY	FRIDAY	SATURDAY	SUNDAY
First Course		Semolina and cheese cream	Courgette cream	Vegetable cream	Semolina and cheese cream	Pumpkin cream	Green beans cream	Carrots cream
Second Course		Egg mousse	Crescenza cheese	Cod mousse with aromas	Fresh cheese	Ricotta cheese	Egg mousse	Robiola cheese
Side Dish		Green beans mousse	Chard mousse	Cauliflower mousse	Purèe	Purèe	Green beans mousse	Courgette mousse
Dessert		Fruit puree	Fruit puree	Fruit puree	Fruit puree	Fruit puree	Fruit puree	Fruit puree
Energy	Kcal	793	776	795	737	587	880	713
Soluble sugar	g	14	14	13	16	28	14	15
Available carbohydrates	g	127	87	128	62	79	140	87
Lipids	g	16	17	14	22	14	10	10
Protein	g	41	24	37	35	34	30	28

TOT		MONDAY	TUESDAY	WEDNESDAY	THURSDAY	FRIDAY	SATURDAY	SUNDAY	Daily Average
Energy	Kcal	1631	1422	1686	1557	1446	1529	1330	1514
Soluble sugar	g	30	35	27	38	43	32	34	34
Available carbohydrates	g	255	160	280	177	226	247	171	217
Lipids	g	32	34	28	44	28	20	20	29
Protein	g	82	48	74	70	68	60	56	65
